# A Novel Multi-Epitope Vaccine Based on Urate Transporter 1 Alleviates Streptozotocin-Induced Diabetes by Producing Anti-URAT1 Antibody and an Immunomodulatory Effect in C57BL/6J Mice

**DOI:** 10.3390/ijms18102137

**Published:** 2017-10-16

**Authors:** Yanjie Ma, Huimin Cao, Zhixin Li, Jinzhi Fang, Xiaomin Wei, Peng Cheng, Rui Jiao, Xiaoran Liu, Ya Li, Yun Xing, Jiali Tang, Liang Jin, Taiming Li

**Affiliations:** School of Life Science & Technology, China Pharmaceutical University, Nanjing 210009, China; 14211030526@stu.cpu.edu.cn (Y.M.); 1621030544@stu.cpu.edu.cn (H.C.); 14211030601@stu.cpu.edu.cn (Z.L.); 2020140639@stu.cpu.edu.cn (J.F.); 13211030585@stu.cpu.edu.cn (X.W.); 15211030512@stu.cpu.edu.cn (P.C.); 15211030561@stu.cpu.edu.cn (R.J.); 14211030603@stu.cpu.edu.cn (X.L.); 1621030486@stu.cpu.edu.cn (Y.L.); xingyun_503@163.com (Y.X.); 13645195250@163.com (J.T.); liangjin1975@cpu.edu.cn (L.J.)

**Keywords:** diabetes, hyperuricemia, urate transporter 1, uric acid, antibody

## Abstract

Hyperuricemia (HUA) is related to diabetes. Uric acid-induced inflammation and oxidative stress are risk factors for diabetes and its complications. Human urate transporter 1 (URAT1) regulates the renal tubular reabsorption of uric acid. IA-2(5)-P2-1, a potent immunogenic carrier designed by our laboratory, can induce high-titer specific antibodies when it carries a B cell epitope, such as B cell epitopes of DPP4 (Dipeptidyl peptidase-4), xanthine oxidase. In this report, we describe a novel multi-epitope vaccine composing a peptide of URAT1, an anti-diabetic B epitope of insulinoma antigen-2(IA-2) and a Th2 epitope (P2:IPALDSLTPANED) of P277 peptide in human heat shock protein 60 (HSP60). Immunization with the multi-epitope vaccine in streptozotocin-induced diabetes C57BL/6J mice successfully induced specific anti-URAT1 antibody, which inhibited URAT1 action and uric acid reabsorption, and increased pancreatic insulin level with a lower insulitis incidence. Vaccination with U-IA-2(5)-P2-1 (UIP-1) significantly reduced blood glucose and uric acid level, increased Th2 cytokines interleukin (IL)-10 and IL-4, and regulated immune reactions through a balanced Th1/Th2 ratio. These results demonstrate that the URAT1-based multi-epitope peptide vaccine may be a suitable therapeutic approach for diabetes and its complications.

## 1. Introduction

Hyperuricemia (HUA) is associated with diabetes, and some studies showed that the prevalence of hyperuricemia in diabetic patients is about 25% [1,2]. In addition, several cases have reported in which high serum uric acid induces inflammation [3], and it is an underlying etiological factor for diabetes and metabolic syndrome [4,5]. As no reports have focused on the treatment effect of diabetes and its complications, especially hyperuricemia, further studies are essential in preventing diabetes and its complications through reducing the uric acid level based on previous studies. 

Uric acid (UA) is the terminal product of the metabolic purines pathway [6]. HUA is due to the disorder of purine metabolism and the urate that is excreted in the urine or gastrointestinal tract [7,8]. HUA is an independent risk factor for diabetes and its complications [6]. Several clinical analyses have revealed that hyperuricemia contributes to insulin resistance, hypertension, and dyslipidemia [9,10,11,12,13,14,15]. In return, these diseases always lead to high uric acid [5,11,12]. Uric acid activates the transcription factor ChREBP. ChREBP (Carbohydrate-responsive element-binding protein) is involved in activating genes that encode fatty acid biosynthesis and the enzyme fructokinase in the liver and adipose tissue [16]. Via these mechanisms, high uric acid could promote obesity and diabetes. Moreover, in diabetic patients, a high level of UA and hyperglycemia induce oxidative stress, which causes serious cellular dysfunction [17]. In our previous studies, streptozotocin (STZ)-induced diabetic mice always had a high uric acid level.

Soluble uric acid, usually forms as the ion form of urate in the blood. URAT1 (urate transporter 1, encoded by *SLC22A12*) in the kidney, is an important urate–anion exchanger, and regulates the blood urate level. About 90% of all soluble UA is filtered through the glomerulus in the kidney, and is eventually re-absorbed to the blood [18,19]. Soluble uric acid is the target of uricosuric and anti-uricosuric agents like benzbromarone, which could promote the excretion of uric acid and thereby lower SUA [20]. 

The multi-epitope vaccine UIP-1 (U-IA-2(5)-P2-1) is a peptide that contains a B-cell epitope antigen from URAT1 and the adjuvant peptide IA-2(5)-P2-1. IA-2(5)-P2-1 consists of P277 peptide Th2 epitope [21] and B-cell insulinoma antigen (IA-2), and it is effectual to prevent the development of type 1 diabetes mellitus (T1DM) [22]. Previous studies confirmed that it is effective at attenuating this T1DM through restoration of the delicate Th1/Th2 balance in animals [23,24]. Piquer et al. [22] demonstrated that residues (626FEYQD630) contain the five amino acids in IA-2 (insulinoma antigen) we named IA-2(5), which are major B cell epitopes of auto-antibodies. Th2 epitope P2 (448IPALDSLTPANED460) [25] is the C-terminal sequence in P277. P277 (437 VLGGGCALLRCIPALDSLTPANED460) is one of the heat shock protein 60 (HSP60) determinants, and could control the incidence of T1DM [26]. 

The present study aims to investigate the function of UIP-1 with high uric acid in STZ-induced diabetes in male C57BL/6J mice. We detected the serum uric acid level and blood glucose. In addition, we further investigated the pancreatic island and URAT1 antibody. 

## 2. Results

### 2.1. Hypoglycemic Activity in Streptozotocin (STZ)-Induced Diabetes in C57BL/6J Mice

**Figure 1:**
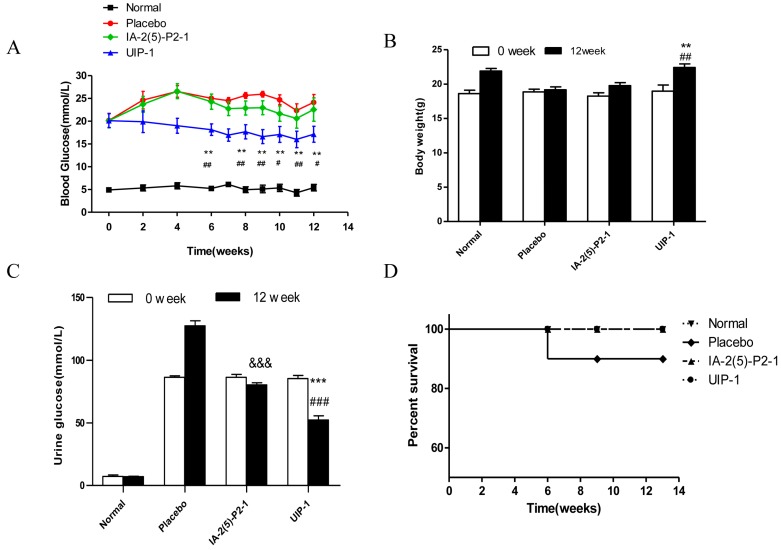
Glucose metabolism was improved by the novel UIP-1 (U-IA-2(5)-P2-1) peptide vaccine in C57BL/6J mice. (**A**) Eight-hour fasting blood glucose concentrations were monitored by glucometers in C57BL/6J mice (*n* = 10 for each group); (**B**) The mean body weight throughout the observation period (*n* = 10 for each group); (**C**) The varieties of urine glucose; (**D**) The survival time of each group was recorded in the study. Values are expressed as the mean ± SEM of data. ** *p* < 0.01, *** *p* < 0.001; UIP-1 versus placebo group. ^#^ *p* < 0.05, ^##^ *p* < 0.01, ^###^ *p* < 0.001; UIP-1 versus IA-2(5)-P2-1 group. ^&&&^ *p* < 0.001; IA-2(5)-P2-1 versus placebo group, one-way ANOVA.

Blood glucose and weight are important indicators of diabetes. We examined the effects of UIP-1 diabetic treatment through blood glucose, weight, urine glucose, and survival rate. Blood glucose and weight were measured weekly in all groups. As shown in Figure 1A, after three weeks of being given the vaccine, the UIP-1 group began to show lower blood glucose; the function lasted from three weeks to the end of the experiment, and the average blood glucose was nearly 16 mmol/L in the end. Placebo mice kept up high blood glucose from the first week to the last. Meanwhile, the UIP-1-treated mice could maintain their normal weight compared with the IA-2(5)-P2-1 and placebo mice (*p* < 0.05) in the process of study (Figure 1B). At the end of the observation period, the urine glucose value was lower than in the placebo and the IA-2(5)-P2-1 group (Figure 1C). In the sixth week, there was a mouse dead in the placebo group because of high blood glucose. Other groups’ mice lived at the end of the experiment (Figure 1D). The results showed that the UIP-1 vaccine significantly alleviated the symptoms of diabetes in STZ-induced diabetic mice.

### 2.2. The Detection of Serum and Urine Uric Acid

**Figure 2:**
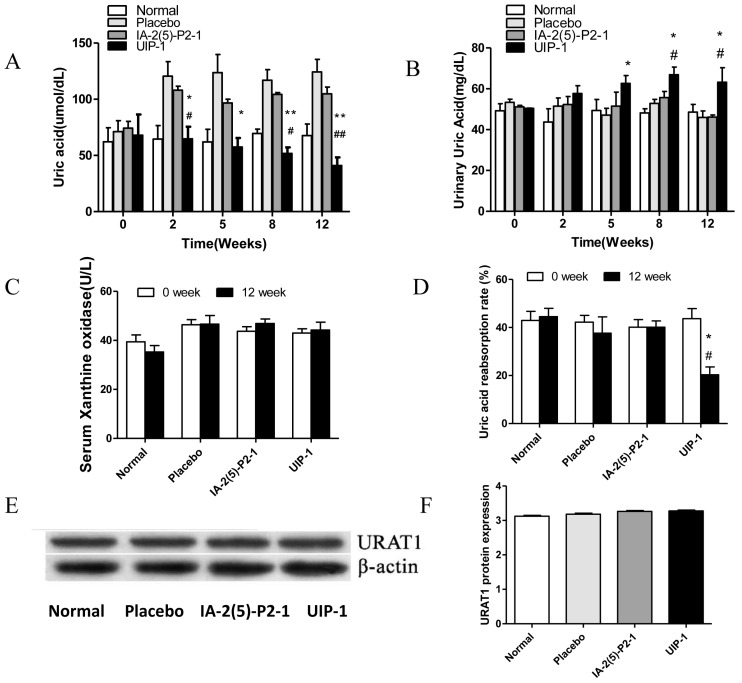
Effect of UIP-1 on the uric acid metabolism. (**A**) The level of serum uric acid; (**B**) The level of urine uric acid; (**C**) The concentration of serum xanthine oxidase; (**D**) Determination of uric acid absorption rate. (**E**,**F**) WB analysis was used in kidney tissue to quantify the expression of URAT1 proteins. Values are expressed as the mean ± SEM of data. * *p* < 0.05, ** *p* < 0.01, UIP-1 versus placebo group. ^#^ *p* < 0.05, ^##^ *p* < 0.01, UIP-1 versus IA-2(5)-P2-1 group, one-way ANOVA.

A high level of uric acid is the most prominent feature of HUA. We detected the level of the serum and urine uric acid and the uric acid reabsorption rate. Then, we investigated the quantity of serum xanthine oxidase (a key enzyme that catalyzes xanthine into uric acid) and the expression of URAT1. Both are important factors determining the UA level. As shown in Figure 2A, the level of SUA in the UIP-1 group was significantly lower compared with the placebo and IA-2(5)-P2-1 groups (*p* < 0.05). On the contrary, the level of urine uric acid (UUA) in the UIP-1 group was significantly higher compared with the placebo and IA-2(5)-P2-1 groups (*p* < 0.05) (Figure 2B). This suggested that the kidney may discharge excess uric acid in the UIP-1 group. The quantity of serum xanthine oxidase in every group was not significantly different (Figure 2C). These data showed that UIP-1 did not affect the expression of xanthine oxidase. The UIP-1 showed a lower uric acid reabsorption rate than the placebo and IA-2(5)-P2-1 groups (Figure 2D). The quantity of the protein URAT1 in every group was not significantly different (Figure 2E). This showed that UIP-1 only inhibited URAT1 and did not affect its expression.

### 2.3. Effect of U-IA-2(5)-P2-1 (UIP-1) on Total Antioxidant Capacity (T-AOC) and Superoxide Dismutase (SOD) Activity and Malondialdehyde (MDA)

**Figure 3:**
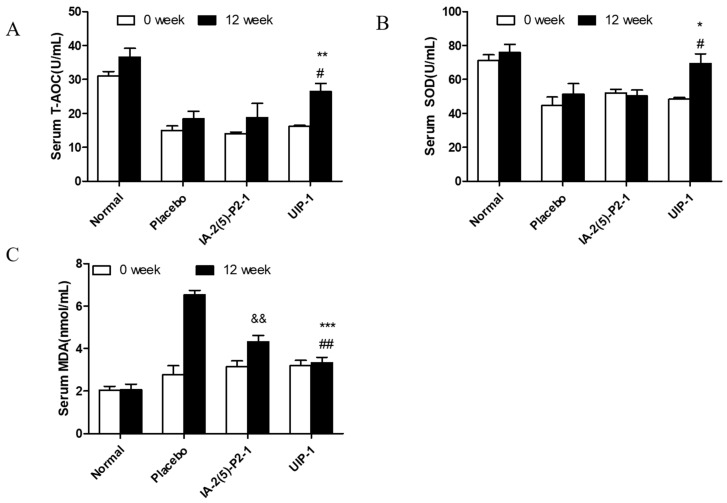
Effect of UIP-1 on serum SOD and T-AOC activity and MDA concentrations in the serum of mice. T-AOC (**A**), SOD (**B**) and MDA (**C**) levels in every group were determined. Data are expressed as the mean ± SEM (*n* = 3). * *p* < 0.05, ** *p* < 0.01, *** *p* < 0.001; UIP-1 versus placebo group. ^#^ *p* < 0.05, ^##^ *p* < 0.01; UIP-1 versus IA-2(5)-P2-1 group. ^&&^ *p* < 0.01; IA-2(5)-P2-1 versus placebo group, one-way ANOVA.

To assess the effects of UIP-1 on oxidative stress responses in STZ-induced diabetes, superoxide dismutase (SOD) and total antioxidant capacity (T-AOC) activity and malondialdehyde (MDA) was detected in the serum. In general, a high level of SUA is characterized by excessive oxidative stress, which damages the β-cell. SOD and T-AOC are antioxidant enzymes that are inactivated by reactions involving ROS and membrane phospholipids that form MDA. At the end of the experiment, the UIP-1 group was significantly different compared to the placebo in T-AOC (Figure 3A), SOD activity levels (Figure 3B), and MDA (Figure 3C) level in the final week. 

### 2.4. Vaccine UIP-1 Protecting Islets from Damaging

**Figure 4:**
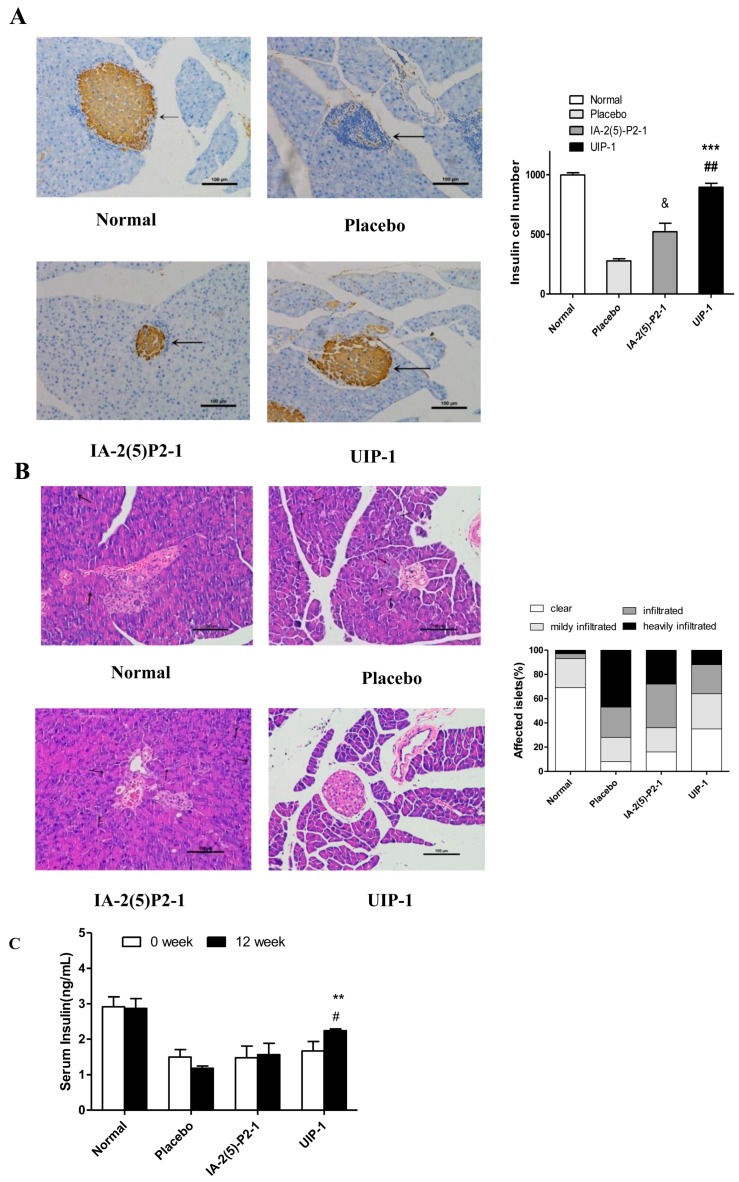
Histological examination of pancreas tissue. (**A**) Immunohistochemistry analysis of insulin with an anti-mouse insulin antibody in islets (200×) The black arrows showed the pancreatic island of mice; (**B**) HE staining of islets from pancreas tissues. The black arrows showed the inflammatory cells. Photomicrographs of representative islets from pancreas tissue (200×); (**C**) The level of serum insulin. Data are expressed as the mean ± SEM (*n* = 3). ** *p* < 0.01, *** *p* < 0.001; UIP-1 versus placebo group. ^#^ *p* < 0.05, ^##^ *p* < 0.01; UIP-1 versus IA-2(5)-P2-1 group. ^&^ *p* < 0.05; IA-2(5)-P2-1 versus placebo group, one-way ANOVA.

Type 1 diabetes (T1DM) is a progressive autoimmune disease caused by the destruction of insulin secreting β-cells by T cells. Insulin, a hormone protein, is produced by the pancreatic islet β cells. It is the only drug used to treat T1DM in a clinical setting. So the pancreata were histologically analyzed at the end of the experiment. The UIP-1 group showed a significant difference to the placebo and IA-2(5)-P2-1 groups in the number of islet cells (Figure 4A). The shape of the islet in the UIP-1 group is clearer, and it had fewer necrosis areas and less lymphocyte infiltration (Figure 4A). The placebo exhibited significant insulitis, indistinguishable islet mass, and infiltration of inflammatory cells, while the UIP-1 group showed normal islet architecture and slight infiltration of inflammatory cells with slight insulitis (Figure 4B). In Figure 4C, the serum levels of insulin were detected. The UIP-1 group was significantly different from IA-2(5)-P2-1 and placebo (*p* < 0.01).

### 2.5. The Detection of URAT1 Antibody and IgG Isotype

**Figure 5:**
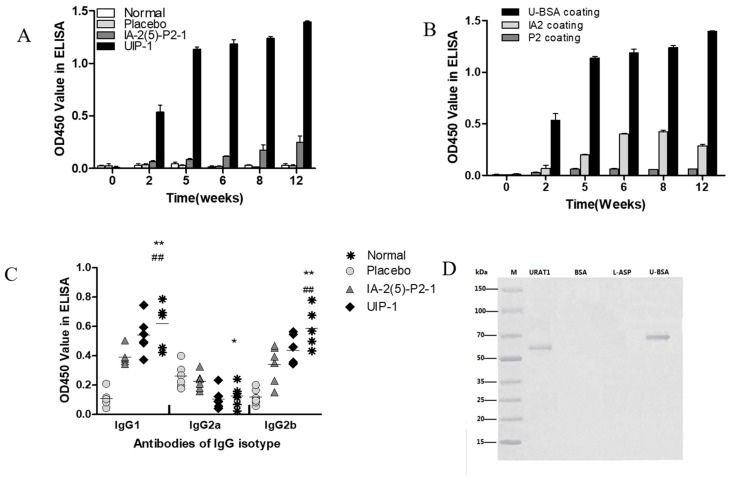
Induction of specific anti-URAT1 antibody after immunization in male C57BL/6J mice. (**A**) Male diabetic mice were immunized with UIP-1 (*n* = 10), IA-2(5)-P2-1 (*n* = 10), or Lipofundin (*n* = 10), as indicated above. The antibody titer is expressed as the mean OD450 value ± SEM; (**B**) Antibodies induced by different epitopes in the multi-epitope peptide UIP-1 were identified using relevant coating antigens in ELISA (*n* = 6/10); (**C**) Antibody isotypes induced by the different vaccines were determined by ELISA (*n* = 6/10 for each group); (**D**) At week 12, sera were collected from UIP-1-immunized mice. Antibodies that specifically recognized and bound to recombinant URAT1 (lane 1) and BSA-conjugated B-cell epitope peptide (lane 4) were detected by Western blotting. BSA (bovine serum albumin) (lane 2) and Asparaginase (lane 3) were not recognized by the antibodies. All data are expressed as the mean ± SEM. * *p* < 0.05, ** *p* < 0.01 (UIP-1 vs. placebo group). ^##^ *p* < 0.01 (UIP-1 vs. IA-2(5)-P2-1 group).

In order to investigate the mechanism of vaccine UIP-1, we examined the quantity and affinity of antibodies. The URAT1 antibody was produced as early as three weeks following the initial inoculation in mice immunized with UIP-1, while mice immunized with IA-2(5)-P2-1 and Lipofundin failed to elicit URAT1 antibody formation (Figure 5A). Then we further investigated the affinity of the URAT1 antibody. In Figure 5B, the ELISA binding assay result showed that most of the antibodies are combined with U-BSA (the U epitope) in the UIP-1 group. Also, the URAT1 antibody recognized recombinant URAT1 protein in the UIP-1 serum by Western blot analysis (Figure 5D).

The level of antibody isotype (IgG1, IgG2a, IgG2b) is a response to the Th1 and Th2 cell reaction, and may be important in mounting antibody responses to vaccination. UIP-1 group shows a significant difference to the placebo group in IgG1, IgG2b, and IgG2a (Figure 5C), and a significant difference to the IA-2(5)-P2-1 group in terms of IgG1 (Figure 5C). It showed that UIP-1 as a vaccine induced a Th2 response, and the vaccine UIP-1 can induce an immune reaction.

### 2.6. T Cell Proliferation and Cytokine Assay

**Figure 6:**
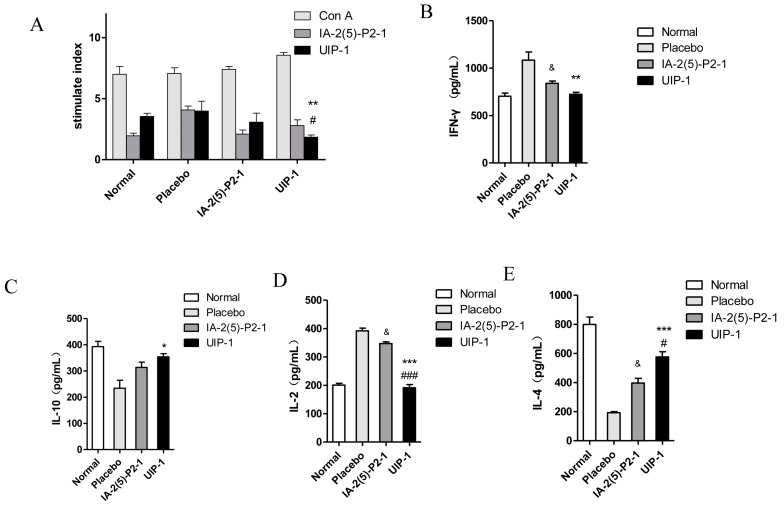
The effects of UIP-1 on immune responses in C57BL/6J mice. UIP-1 administration inhibited determinant spreading of T cell autoimmunity. At week 12 of the experiment, splenic T cells from the UIP-1 group, IA-2(5)-P2-1 group, and placebo were tested for proliferative responses to UIP-1, IA-2(5)-P2-1, and Concanavalin A (Con A). Data are expressed as the mean ± SEM stimulation index (SI) of triplicate samples (*n* = 3/9). (**A**) The T cell proliferation in every group. The level of IFN-γ (**B**), IL-10 (**C**), IL-2 (**D**), IL-4 (**E**) after stimulated as above. * *p* < 0.05, ** *p* < 0.01, *** *p* < 0.001; UIP-1 versus placebo group. ^#^ *p* < 0.05, ^##^ *p* < 0.01, ^###^ *p* < 0.001, UIP-1 versus IA-2(5)-P2-1 group, ^&^ *p* < 0.05; IA-2(5)-P2-1 versus placebo group, one-way ANOVA.

Splenic T cells were tested for their proliferative ability given extra immunization during the last week. This reflects the vaccine’s function on regulation of the immune system. For UIP-1 group, there was no significant difference between IA-2(5)-P2-1- and UIP-1-stimulated splenocytes’ proliferation (Figure 6A). However, Concanavalin A (Con A)-stimulated splenocytes’ proliferation was greater than IA-2(5)-P2-1 and UIP-1 stimulated splenocytes’ proliferation. These results showed that UIP-1 can downregulate spontaneous proliferative T cell responses, which conforms to the treatment mechanism of type 1 diabetes.

To test whether UIP-1 will enhance the Th2-like immune response by multi-point subcutaneous injection, we examined cytokines’ expression in spleen cells using a cytokines assay kit. The results showed a decreased pro-inflammatory cytokine of IFN-γ and IL-2 (Figure 6B,D) and increased anti-inflammatory cytokines of IL-10 and IL-4 (Figure 6C,E) in the animals in the UIP-1-treated group compared with the placebo (*p* < 0.01). 

## 3. Discussion

Diabetes is prevalent in the modern world, and many diabetes complications are attracting more and more attention. High blood glucose always leads to tissue damage, especially of capillary endothelial cells and mesangial cells [27]. Hyperuricemia and renal failure are the main complications in diabetes [28]. Renal dysfunction can be fatal for diabetic patients [29]. Hyperuricemia and hyperglycemia are the main reasons for renal failure [30]. Diabetic nephropathy, hypertension, and insulin resistance can impede renal tubular excretion, which simultaneously increases the level of uric acid. Hyperuricemia is a purine metabolic disorder disease that is associated with metabolic syndrome and diabetes. The principal reason is the excessive formation of uric acid or a decrease in renal excretion [31]. Hyperuricemia is correlated with obesity, hyperlipidemia, hypertension, diabetes, atherosclerosis, and other diseases [32]. In addition, a high level of serum uric acid often causes gout, kidney stones, and kidney disease through monosodium urate (MSU), a uric acid crystal [33]. It is recognized by toll-like receptors that activate the NALP3 inflammasome [34]. Serum uric acid also causes diabetic patients to have cardiovascular complications and increases the cardiovascular end point event rate [35].

Base on previous studies, we put forward the hypothesis that URAT1-based combinatorial peptide reduces the level of urate, and we further found that it induces a beneficial immunomodulation in diabetic mice. The UIP-1 group could reduce blood glucose and the serum uric acid level by producing the URAT1 antibody (and apparently no other antibody) through inhibiting uric acid re-absorption and regulating the immune system (Figure 2). 

Experimental data revealed that the UIP-1 vaccine can effectively reduce high blood glucose (Figure 1A), and decreased the serum uric acid level in male C57BL/6J mice (Figure 2A) as well. In addition, it serves as a therapeutic target for suppressing diabetes and its complications through regulating systemic immune responses. The vaccine UIP-1 (U-IA-2(5)-P2-1) is a multi-epitope vaccine, and includes a peptide of IA-2(5)-P2-1 and a U epitope to reduce uric acid. In other words, UIP-1 consists of a B-cell epitope of URAT1, IA-2 [22], and a Th2 epitope peptide of P277 [21]. The results show that the therapeutic mechanism may be to decrease uric acid through UIP-1 stimulating the animal to produce URAT1 antibody (Figure 5), which can inhibit URAT1 uric acid re-absorption. To get a full appreciation of URAT1 antibody, we analyzed the IgG isotype from the mice serum. When C57BL/6 mice were immunized with the UIP-1 vaccine, they developed sufficient levels of IgG1 and IgG2b (Figure 5C) antibody responses compared to the IA-2(5)-P2-1 and placebo groups, giving evidence of Th2-mediated immunity. This indicated that UIP-1 tended to treat a Th2-type predominant immune response. This type of antibody could balance the Th1/Th2 ratio. T1DM is an autoimmune disease, and one of its mechanisms is the imbalance of the Th1/Th2 ratio, which leads to the immune system attacking islet cells [36,37,38]. Apparently, the URAT1 antibody may have hypouricemic and hypoglycemic activity, protecting the β-cell from damage by avoiding oxidative stress (Figure 3) and reducing inflammation (Figure 6). 

The therapeutic mechanism was based on the URAT1 antibody, for it could decrease uric acid, which can cause inflammation and oxidative stress. Urate transporters in the kidney such as URAT1, GLUT9 (glucose transporter 99), and OAT1 (organic anion transporter 1) play important roles in the urate excretion [39,40,41], and are important targets for drugs to treat hyperuricemia. Allopurinol, benzbromarone, and febuxostat are common drugs to treat hyperuricemia. All of them are chemical drugs that can reduce serum uric acid. Some studies reported that this drug also alleviates diabetes [42,43,44,45]. Benzbromarone, sulfinpyrazone, and probenecid are URAT1 inhibitors that restrict uric acid reabsorption. It has been reported that benzbromarone could lower the level of blood glucose in *db*/*db* mice and inhibit human fatty acid binding protein 4 [41,46]. However, all of them are chemical drugs that can lead to allergies and many side effects [47,48]. They have been demonstrated to produce adverse effects such as hypersensitivity and agranulocytosis [49]. Therefore, hypouricemic patients need safer and more effective anti-hyperuricemic agents. 

Patients with diabetes mellitus are at a greater risk of death. This risk seems to be modulated by kidney dysfunction [26]. High uric acid could induce endothelial dysfunction, oxidative stress, and inflammation in the kidney [50]. Hyperuricemia and hyperglycemia are interreactions that interfere with the balance of organism metabolism, thereby increasing the risk of death from diabetes mellitus. Compared to the IA-2(5)-P2-1-treated group, the UIP-1-treated group showed a significant difference in blood glucose (Figure 1A) and SUA level. The results showed that, compared with IA-2(5)-P2-1, UIP-1 can stimulate mice to produce URAT1 antibody (Figure 5). We further observed URAT1 protein and serum xanthine oxidase expression, there was not a significant difference among the groups (Figure 2C,E). The results showed that the novel multi-epitope vaccine UIP-1 can induce mice to produce anti-URAT1 antibody, and can also inhibit uric acid re-absorption and reduce the level of serum uric acid, through which it can suppress inflammation and improve inflammation-related diseases.

Type 1 diabetes is a progressive autoimmune disease caused by the destruction of insulin secreting β-cells by T cells [51]. The typical pathological features of T1DM is chronic inflammation and a Th1/Th2 imbalance [37,38]. Compared with IA-2(5)-P2-1 and the placebo group, the UIP-1 group stimulated immune responses, reduced IFN-γ and IL-2 cytokine (Figure 6B,D), and increased Th2 cytokines IL-10 and IL-4 (Figure 6C,E). The results showed that UIP-1 induces a Th1/Th2 balance, which can protect the pancreas. Because of this, the UIP-1 group can produce more insulin than the IA-2(5)-P2-1 group, and the UIP-1 group shows lower blood glucose than IA-2(5)-P2-1 and the placebo.

Generally, the low immunogenicity of peptide vaccines restricts the use of peptide vaccines. However, UIP-1 contains the peptide Th2 antigen epitope, and ELISA (Figure 5) showed that UIP-1 can introduce enough URAT1 antibody. The results showed that UIP-1 is a qualified antigen that can stimulate mice to produce URAT1 antibody (Figure 5A,B). We are making the URAT1 monoclonal antibody in our laboratory to further analyze its function. URAT1 encoded by SLC22A12 has 553 amino acids, located at the apical membrane of the proximal tubules, that play important roles in the regulation of serum uric acid [52]. It should be noted that this study has examined only the level of SUA and the quantity of URAT1-antibody. It is hard to analyze the mechanism of the inhibition. The three-dimensional structure of URAT1 is uncertain, and there are no documents about the crystal structure of URAT1.

In summary, this study has indicated that UIP-1 has hypouricemic and hypoglycemic activities, and it is a novel multi-epitope vaccine based on URAT1 antibody. We found that it decreased serum uric acid and blood glucose through URAT1 antibody, protected pancreatic islets from damage, and suppressed inflammation and oxidative stress. These effects of UIP-1 are correlated with introducing URAT1 antibody and decreasing levels of inflammatory factors. Results from the present study provide further insights into the effects of UIP-1 on improving TIDM and reducing the level of SUA and anti-inflammatory response, which may lead to a development of effective therapeutic measures to combat TIDM or hyperuricemia. There are hopes that the problem of T1DM will become manageable.

## 4. Materials and Methods

### 4.1. Vaccine Design and Synthesis

The peptide was constructed and synthesized through FMOC solid phase synthesis in Shanghai glbiochem Co., Ltd. (Shanghai, China). Synthetic peptides were purified by reverse-phase HPLC (>93% purity). IA-2(5)-P2-1 peptide contains a B-cell epitope of IA-2 (insulinoma antigen) we named IA-2(5) and a Th2-cell epitope of P277 we named P2. The C terminal of the B-cell epitope peptide of URAT1 was conjugated to the combinatorial peptide IA-2(5)-P2-1. The new multi-epitope peptide was named UIP-1 (also named as U-IA-2(5)-P2-1). 

### 4.2. Mice

Four-week-old male C57BL/6J mice were bought from the Jiangsu Experimental Animal Center (Yangzhou Animal Center, Yangzhou, China) and housed under SPF conditions. The mice were kept in a separate room at the following conditions: 24 ± 2 °C, 50 ± 10% relative humidity, 12/12 h light–dark cycle, and free access to food and water. The studies were approved by the animal ethics committee of China Pharmaceutical (approval No. CPUSPF/SQ-16025, 12, February, 2016). After two weeks of acclimatization to our animal house, animals received a dose of STZ (50 mg/kg) (Sigma, St. Louis, MO, USA) for five consecutive days intraperitoneally. Blood glucose was measured at regular intervals using an automatic glucose analyzer (GlucoLeader, Taiwan) to determine the development of diabetes after two weeks. The diabetic mice models were judged to have a blood glucose concentration ≥11.1 mmol/L. After two weeks, the diabetic mice were divided into three groups based on their last blood glucose reading, and we then injected the vaccine. 

### 4.3. Animal Experiments

The diabetic mice were divided into three groups of 10 mice each. Normal animals (*n* = 10) served as controls. The first group was treated with a subcutaneous injection of 100 µg of UIP-1. The vaccine of UIP-1 was solubilized in Lipofundin (B. Braun, Melsungen, Germany) with 4 mg mannitol; Lipofundin served as an adjuvant. The second group was treated with a subcutaneous injection of 100 µg of IA-2(5)-P2-1. The vaccine of IA-2(5)-P2-1 was solubilized, the same as UIP-1. The third group was immunized with Lipofundin (B. Braun, Melsungen, Germany) with 4 mg mannitol in accordance with the immunization procedure above. Groups of mice were injected at 0, 1, 2, 3, 4, 6, 8, 10, and 12 weeks. Weight and 8-h fasting blood glucose levels were measured using an automatic glucose analyzer (GlucoLeader, Taiwan) before every inoculation. Serum samples were collected every week and stored at −20 °C.

### 4.4. ELISA Analyses of Specific Antibodies and the Relative Affinity of Antibodies against URAT1

In order to detect and evaluate the URAT1-specific antibodies, enzyme-linked immunosorbent assay (ELISA) was used. A 96-well ELISA plate was coated with 100 μL U-BSA (Shanghai glbiochem Co., Ltd., Shanghai, China), which was diluted with 0.1 mol/L carbonate bicarbonate buffer (pH = 9.5) and kept overnight at 4 °C. Wells were blocked with PBS (pH = 7.4) containing 5% (*w*/*v*) BSA (Sigma, St. Louis, MO, USA) for an hour [53]. Then, we washed the ELISA plate and added serum samples. Each well was incubated with 1:100 dilutions of serum samples for 2 h. Horseradish peroxidase-conjugated goat anti-rat IgG was added to detect the binding antibodies [54] (Transgen Biotech, Beijing, China). The wells were washed three times with PBST. To each well, we added 100 μL TMB Horseradish Peroxidase Color Development Solution for ELISA (Beyotime, Nanjing, China) for 30 min at 37 °C. The reaction was halted by adding 50 µL 2 M H_2_SO_4_. Each well was assayed in an ELISA plate reader (KHBST-360, Shanghai, China). Each serum was verified in triplicate at OD 450 nm. The method for ELISA was that described by Jin et al. [26,54]. 

In the UIP-1 group, we analyzed the relative avidity of antibodies against URAT1. Briefly, a 96-well ELISA plate was coated with 100 µL of U-BSA, IA2-BSA, and P2-BSA (Shanghai glbiochem Co., Ltd., Shanghai, China), diluted with 0.1 M carbonate bicarbonate buffer (pH = 9.5) and kept overnight at 4 °C. The following step was similar to the ELISA method mentioned above.

### 4.5. Urine Glucose Tests

The mice’s urine was collected at a fixed period of time in mice metabolic cages. Urine glucose was tested using Glucose oxidase/peroxidase reagent (Shanghai Rongsheng Biotech Co., Ltd., Shanghai, China). Glucose is oxidized to gluconic acid and H_2_O_2_ through glucose oxidase. H_2_O_2_ reacts with *O*-dianisidine to form a colored product. Adding H_2_SO_4_ causes a reaction with oxidized *O*-dianisidine to form a more stable colored product. The concentration of the pink color measured at OD 540 nm is proportional to the original glucose concentration. The operation steps followed the instructions.

### 4.6. Antibody Isotyping Analyses

Antibody isotyping analysis: IgG1, IgG2a, IgG2b were detected by antibody isotyping ELISA kits (Meimian Biotech Co., Ltd., Yancheng, China). The kits were used to determine the IgG subclass in serum samples at the end of the experiment. The experimental process was performed according to the manufacturer’s instructions. Briefly, 0.1 mL serum was incubated for 30 min at 37 °C in a 96-well ELISA plate. After the plates were washed, the binding of antibodies was detected using horseradish peroxidase-conjugated goat anti-rat IgG (Transgen Biotech, Beijing, China). The reaction was stopped by 2 M H_2_SO_4_ and the samples were read at OD 450 nm by a multi-scan spectrum microplate spectrophotometer (KHBST-360, Shanghai, China) at room temperature.

### 4.7. Uric Acid Assay

The serum level of uric acid was determined by a uric acid detection kit (Nanjing Jiancheng Biotech, Nanjing, China). The experimental process was performed according to the manufacturer’s instructions. Urine uric acid was determined as per the serum uric acid detection. The experimental process was performed according to the manufacturer’s instructions.

### 4.8. Uric Acid Reabsorption Rates

The RTECS (renal tubular epithelial cells) were isolated from two mice in each group. The RTECS were counted and suspended in 10% FBS containing DMEM medium. The cells’ density was 10^5^/mL. Then we put the RTECS suspension into a 96-well plate, where each hole is 100 μL. The 96-well plate was placed in a cell culture incubator (Thermo Finnigan, San Jose, CA, USA). One hundred microliters of uric acid solution containing the same concentration for each group were added to the 96-well plate. The final concentration of uric acid solution is 0.05 mol/L. After three hours in the cell culture incubator, the culture supernatant’s uric acid (SuUA) was determined. The uric acid was determined by a uric acid detection kit (Nanjing Jiancheng Biotech, Nanjing, China). The calculation of the uric acid absorption rate was as follows: Uric acid reabsorption rate = (50 − SuUA)/50 × 100%. 

### 4.9. T Cell Proliferation Assays

One week after the last administration, mice spleens were removed and the T cell proliferative responses were assayed in vitro. The method was that described by Jin et al. [54]. Briefly, a microtiter plate contained 2 × 10^5^ cells in 0.1 mL of RPMI-1640 culture medium in triplicate in the presence of Con A, IA-2(5)-P2-1, and UIP-1. Dose–response curves were made to establish optimal doses. The concentration of 5 µg/mL was chosen for IA-2(5)-P2-1 and UIP-1 and 1.25 µg/mL was chosen for Con A. Cultures were incubated for 72 h at 37 °C in a humidified atmosphere with 5% CO_2_. T cell responses were observed by the MTT method. Briefly, 0.02 mL of MTT (Sigma, St. Louis, MO, USA) solution (5 mg/mL in PBS) was added to each well, and the microplates were further incubated at 37 °C for 4 h in a humidified atmosphere with 5% CO_2_. The collected cells’ supernatants were centrifuged for cytokine assay and 100 μL DMSO (Dimethyl Sulphoxide) (Sigma, St. Louis, MO, USA) was added to dissolve the formazan crystal. Formazan was quantified by a multiskan spectrum microplate spectrophotometer (KHBST-360, Shanghai, China) with a 570 nm test wavelength. Data were expressed as mean stimulation index (SI) of triplicate sample ± SEM. Stimulation Index (SI) = the average absorbance value of the test group/the blank group (culture medium as the blank group).

### 4.10. SDS-PAGE and Western Blot

Ten micrograms of protein from each sample were separated by 10% SDS-PAGE and transferred to PVDF membranes (Biosky Biotech Co., Ltd., Nanjing, China). After blocking in 5% non-fat dry milk, which was dissolved in TBST at room temperature for 1 h, PVDF (polyvinylidene difluoride) membranes were incubated with URAT1 and β-actin antibody (1:2000) overnight at 4 °C. Membranes were washed with PBST and incubated with horseradish peroxidase-conjugated secondary antibody (1:5000) for an hour.

The detection of URAT1 antibody was as above. After transferring the proteins URAT1, U-BSA, BSA, and Asparaginase to PVDF membranes, the membranes were incubated overnight at 4 °C in UIP-1 mice serum (dilution 1:200). 

The immunoreactive complexes were visualized by the ECL A test and ECL B test kit (Aogene Biotech Co., Ltd., Nanjing, China). Actin quantification served as an internal standard to correct for variations in total protein loading. The luminescent signals were measured by Bio-rad ChemiDoc XRS+ System (Hertfordshire, UK) and densitometry quantification was performed using the AlphaEase software (AlphaInnotech, San Leandro, CA, USA).

### 4.11. Cytokine Assays 

We assayed the splenocytes isolated from IA-2(5)-P2-1, UIP-1 and placebo-treated animals to check their proliferative response to IA-2(5)-P2-1, UIP-1, and Con A. The method was that described by Jin et al. [54]. Culture supernatants were collected after T cells’ proliferation and circulating levels of IL-2, IL-4, IL-10, and IFN-γ were measured in serum using ELISA kits (Aogene Biotech Co., Ltd., Nanjing, China). The experimental process was performed according to the manufacturer’s instructions. Biosource recombinant mouse cytokines were used as standards for calibration curves. The steps were similar to the ELISA method mentioned above. Briefly, 0.1 mL blood supernatants or recombinant cytokine was incubated for 2 h at 37 °C. After the plates were washed, 0.1 mL biotinylated detection antibodies was added and the plates incubated for 1 h at 37 °C, then extensively washed and incubated with streptomycin conjugated to alkaline phosphatase for 1 h at 37 °C. After washing, alkaline phosphate substrate was added to each well and incubated at 37 °C for 10 min in a dark room. The reaction was halted by 100 μL 2 M H_2_SO_4_ and the samples were read at 450 nm by a multi-scan spectrum microplate spectrophoto meter (KHBST-360, Shanghai, China) at room temperature. Cytokine levels are expressed as picograms per milliliter based on calibration curves. The lower limit of detection for the experiments described in this paper was 15 pg/mL for cytokines. 

### 4.12. Measurement of SOD, T-AOC, and MDA Activity 

SOD and T-AOC activity were measured using commercially available assay kits (Nanjing Jiancheng Biotech, Nanjing, China), according to the manufacturer’s instructions. MDA levels were measured using a thiobarbituric acid reactive substances assay kit (Nanjing Jiancheng Biotech, Nanjing, China), according to the manufacturer’s instructions.

### 4.13. Serum Insulin Determination

Serum insulin was detected by insulin determination ELISA kits (Meimian Biotech Co., Ltd., Yancheng, China). The kits were used to determine the insulin in serum samples. The experimental process was according to manufacturer’s instructions. The process was the same as for the ELISA analyses mentioned above.

### 4.14. Histological Analysis and Immunohistochemical Analysis

The pancreata were fixed with 10% formalin solution and embedded in paraffin. The pancreas was sectioned to a thickness of 3 μm for hematoxylin and eosin (H & E) staining. The process was followed as described by Lu et al. [55]. The average degree of insulitis was assessed over 20 islets scored per pancreas. The degree of insulitis was classified as follows: no insulitis (without cell infiltration); peri-insulitis (infiltrated occupying < 25% of the islet mostly around them); mild insulitis (25% ≤ infiltrated occupying < 50% of the islet area); severe insulitis (cell infiltrated occupying ≥ 50% of the islet area). 

Islet cells were analyzed by immunohistochemistry. Sections were stained with anti-insulin antibodies (Servicebio Biotech Co., Ltd., Nanjing, China), then observed under a laser scanning confocal microscope.

### 4.15. Statistics

All data are presented as mean ± standard error of mean (SEM). Statistical comparisons were evaluated by one-way ANOVA test and Turkey HSD using SPSS software (ver. 19.0, IBM, Armonk, NY, USA) if the data were normal distributions such as blood glucose, body weight, etc. If the data were non-normal distribution, we used the Kruskal–Wallis test.

## Abbreviations

BSA	Bovine serum albumin
ChREBP	Carbohydrate-responsive element-binding protein
ELISA	Enzyme-linked immunosorbent assay
GLU9	Glucose transporter 9
IA-2	Insulinoma antigen-2
IFN-γ	interferon-gamma
IL-2	Interleukin-2
IL-4	Interleukin-4
IL-10	Interleukin-10
MDA	Malondialdehyde
MTT	Methyltrityl
MSU	Monosodium urate
OAT1	Organic anion transporter 1
PBS	Phosphate-buffered saline
PBST	Phosphate-buffered saline containing 0.1% Tween-20
PVDF	Polyvinylidene luoride
ROS	Reactive oxygen species
RTECS	Renal tubular epithelial cells
SDS-PAGE	Sodium dodecyl sulfate polyacrylamide gel electrophoresis
SOD	Superoxide dismutase
STZ	Streptozotocin
SUA	Serum uric acid
SuUA	Supernatant’s uric acid
T-AOC	Total antioxidant capacity
T1DM	Type 1 Diabetes Mellitus
UIP-1	U-IA-2(5)-P2-1
URAT1	Urate transporter 1
UUA	Urine uric acid
